# Tomatoes

**Published:** 1870-07

**Authors:** 


### TOMATOES

Act medically in two ways, their acid stimulates the liver to work off the surplus bile of summer; the seeds act mechanically upon the bowels, like white mustard seed, or the seeds of figs, and thus tend to prevent constipation and to remove it, while their nutritious qualities make them fit food for the table, whether raw, sliced, fried in sugar, or prepared with bread crumbs; they can be taken with the utmost freedom for breakfast and dinner.

